# A Study of the Correlation of Perfusion Parameters in High-Resolution GRASP MRI With Microvascular Density in Lung Cancer

**DOI:** 10.1002/jmri.26340

**Published:** 2018-11-03

**Authors:** Lihua Chen, Xianchun Zeng, Youli Wu, Xiaochu Yan, Xuequan Huang, Hui Chen, Jiuquan Zhang, Jian Wang, Li Feng

**Affiliations:** 1 Department of Radiology, Southwest Hospital, Army Medical University (Third Military Medical University), Chongqing, P.R. China; 2 Department of Radiology, PLA 101st Hospital, Wuxi Jiangsu, P.R. China; 3 Department of Radiology, Guizhou Provincial People’s Hospital, Guizhou, P.R. China; 4 Department of Pathology, Southwest Hospital, Army Medical University (Third Military Medical University), Chongqing, P.R. China; 5 Department of Radiology, Chongqing University Cancer Hospital & Chongqing Cancer Institute & Chongqing Cancer Hospital, Chongqing, P.R. China; 6 Key Laboratory for Biorheological Science and Technology of Ministry of Education (Chongqing University), Chongqing University Cancer Hospital & Chongqing Cancer Institute & Chongqing Cancer Hospital, Chongqing, P.R. China; 7 Department of Medical Physics, Memorial Sloan Kettering Cancer Center, New York, New York, USA

## Abstract

**Background:**

The histological count of microvascular density (MVD) is the current clinical standard for assessing tumor angiogenesis. Although it is hypothesized that perfusion MRI can be a noninvasive alternative to MVD, there have been few studies to validate their correlations, particularly in lung cancer.

**Purpose:**

To investigate the correlation between MVD and perfusion parameters obtained from high-resolution GRASP (Golden-angle RAdial Sparse Parallel) dynamic contrast-enhanced (DCE)-MRI in a cohort of lung cancer patients, and to validate that GRASP MRI can serve as a free-breathing, noninvasive imaging approach for studying tumor angiogenesis.

**Study Type:**

Prospective.

**Population:**

Twenty-five lung cancer patients (16 male, 9 female, mean age = 57.3 ± 11.7 years).

**Field Strength/Sequence:**

3T MRI; a prototype golden-angle stack-of-stars sequence.

**Assessment:**

Contrast-enhanced MR data were acquired during free breathing and were reconstructed using GRASP with a temporal resolution of ~3 sec/phase. For all data, perfusion analysis was performed using a standard Tofts model to generate the volume transfer coefficient (K^trans^) and the interstitial volume (V_e_). The MVD of corresponding tumor specimens, obtained from Computed Tomography-guided biopsies, were counted with CD34 staining.

**Statistical Tests:**

Pearson correlation analysis; one-way analysis of variance analysis; least significant difference-t method of multiple comparisons.

**Results:**

The correlation coefficient was 0.983 and 0.972 for the measurement and remeasurement of K^trans^ and V_e_. The mean values of K^trans^, V_e_, and MVD were 0.33 ± 0.22 min^−1^, 0.25 ± 0.12, and 49.68 ± 27.08 vessels/0.723 mm^2^, respectively, in all patients (*n* = 25); 0.36 ± 0.26 min^−1^, 0.27 ± 0.13, and 49.09 ± 29.84 vessels/0.723 mm^2^, respectively, in adenocarcinoma (*n* = 15); 0.34 ± 0.17 min^−1^, 0.26 ± 0.12, and 53.85 ± 23.53 vessels/0.723 mm^2^, respectively, in squamous cell carcinoma (*n* = 8); and 0.13 ± 0.15 min^−1^, 0.14 ± 0.06, and 37.20 ± 28.28 vessels/0.723 mm^2^, respectively, in small-cell carcinoma (*n* = 2). There was a positive relationship between the K^trans^ and MVD in all patients (*r* = 0.738, *P* < 0.001).

**Data Conclusion:**

High spatiotemporal resolution DCE-MRI using GRASP is a promising noninvasive alternative to the histological count of MVD for assessing tumor angiogenesis in lung cancer.

**Level of Evidence:**

1

**Technical Efficacy:**

Stage 2

Lung cancer is the leading and increasing cause of cancer-related death among both men and women worldwide.^1^ Due to the vascular dependence of a tumor, tumor angiogenesis plays an important role in the assessment of tumor growth, invasion, and metastasis,^2,3^ as well as in the management of treatment response and prognosis. The current morphological standard for assessing the angiogenesis of a tumor is a histological count of microvascular density (MVD), which reflects tumor angiogenesis and is a biomarker closely associated with the level of lymphatic metastasis, distant metastasis, and histopathological classification.^4^ Therefore, MVD can serve as a surrogate factor in predicting tumor invasiveness and metastasis.^2,3^ However, the histological count of MVD is a complex, cumbersome, and inherently invasive procedure. More importantly, as tumors are normally heterogeneous and the level of angiogenesis is often higher in the peripheral region of the tumor, pathological specimens obtained from a specific local region do not represent the entire tumor, thus leading to potential underestimation or overestimation of the degree of tumor angiogenesis. In addition, without information about vascular permeability, MVD alone cannot determine whether the vessel is hyperpermeable, which is also an important factor associated with tumor characteristics. As a result, a noninvasive technique that can study the angiogenesis of a whole tumor in vivo is highly desirable.

It has been shown that high-resolution dynamic contrast-enhanced magnetic resonance imaging (DCE-MRI) has potential in evaluating tumor angiogenesis and can be an alternative tool to the histological count of MVD.^5^ DCE-MRI is a noninvasive measure that does not require radiation exposure and provides excellent soft-tissue contrast. The status of DCE-MRI in early clinical trials was reviewed and discussed by the Pharmacokinetic and Pharmacodynamic Technologies Committee and Imaging Committee of the Experimental Cancer Medicine Centre,^5–7^ and DCE-MRI has been recommended to assess tumor vascular function and corresponding treatment response. However, despite good potential, there is still a lack of established and standardized methodology for data acquisitions, reconstruction, and image analyses in DCE-MRI.^8^ Moreover, it is in general not trivial to manage the balance between spatiotemporal resolution and volumetric coverage, as well as different types of physiological motion in moving organs such as the lung.

Golden-angle RAdial Sparse Parallel (GRASP) MRI, a relatively new imaging framework combining multicoil compressed sensing with golden-angle radial sampling for rapid and continuous MRI,^9^ has been shown as a promising technique for DCE-MRI.^10^ The radial sampling scheme enables continuous data acquisitions during free breathing; the combination of compressed sensing and parallel imaging enables high temporal resolution and spatial resolution for different dynamic imaging applications; and, more importantly, the golden-angle radial sampling scheme allows a dual-mode reconstruction strategy that can be used for both morphological qualitative assessment and quantitative perfusion analysis simultaneously. The performance of GRASP has been demonstrated in a range of clinical applications,^9,11–13^ and it has recently been extended to free-breathing DCE-MRI of the lung for assessment of pulmonary lesions.^14^ These preliminary studies have all shown the great potential and capabilities of GRASP for improving the usefulness and effectiveness of DCE-MRI towards routine clinical use. However, whether the GRASP technique can serve as an alternative method to the histological count of MVD for studying tumor angiogenesis remains unknown. Therefore, the purpose of the present study was to investigate the correlation between perfusion parameters derived from high-resolution GRASP MRI and microvascular density counted in tumor specimens in a cohort of patients previously diagnosed with different types of lung cancer. The hypothesis was that free-breathing high-resolution DCE-MRI using GRASP can be a useful noninvasive technique to assess tumor angiogenesis and can also be used to monitor treatment response in patients with lung cancer.

## Materials and Methods

### Patient Population

This prospective study was approved by the Institutional Medical Research Ethics Committee. A total of 40 consecutive patients (26 male and 14 female, mean age = 57.1 ± 13.9 years) with known lung lesions confirmed in previous exams were recruited between January 2017 and December 2017. All patients provided informed consent prior to the MRI scan, including the use of their corresponding CT-biopsy and MVD results if available. Patients were recruited only if their clinical history showed at least one primary pulmonary lesion identified in previous exams and the size of the lesion was larger than 15 mm. However, MR data from patients with the following conditions were excluded for use in the current study: 1) patients without pathological biopsy results; 2) patients without corresponding MVD results; 3) patients with benign lesions. These criteria led to a total of 25 patients (16 male, 9 Female, mean age = 57.3 ± 11.7 years) whose data were finally used for our analyses, including 15 patients with adenocarcinomas, 8 patients with squamous cell carcinomas, and 2 patients with small-cell lung cancer. The average size of the lesions in these 25 patients was 4.9 ± 0.9 cm.

### Data Acquisition and GRASP Reconstruction

Lung MRI was performed on a clinical 3T MR scanner (Magnetom TimTrio, Siemens Healthineers, Erlangen, Germany). Data were continuously acquired using a prototype golden-angle stack-of-stars sequence,^15^ and all patients were asked to breathe normally during the entire radial scans. Relevant acquisition parameters were as follows: repetition time / echo time (TR/TE) = 3.40/1.64 msec, field of view (FOV) = 320 × 320 × 120 mm^3^, in-plane matrix = 256 × 256, flip angle = 12° , and in-plane spatial resolution = 1.25 × 1.25 mm^2^. A total of 24 partitions were acquired with a slice thickness of 5 mm, and 3000 spokes were acquired in each partition. A weight-based full dose injection (0.2 mmol/kg) of Magnevist (Bayer Healthcare, Berlin, Germany) was injected in each scan at a rate of 3 mL/sec, ~20 seconds after the start of data acquisition. The scan time of the radial acquisition was 281 seconds for each patient.

GRASP reconstruction was performed using a multicoil compressed sensing framework as previously described^9^ to solve the following cost function:

(1) $$x=\underset{x}{\text{argmin}}\frac{1}{2}{\left\|FCx-y\right\|}_{2}^{2}+\lambda {\left\|Tx\right\|}_{1}$$

Here, *F* is the nonuniform fast Fourier transform (NUFFT) operator, *C* is the coil sensitivities estimated from the artifact-free image reconstructed by averaging all acquired spokes together, *y* is the dynamic multicoil radial *k*-space sorted into different contrast phases, *x* is the corresponding dynamic image series to be reconstructed, and *T* is a sparsifying transform. The L_1_ norm, defined as the sum of absolute values, was used to promote sparsity. The L_2_ norm, defined as the square root of the sum of squares, was used to ensure data consistency (e.g., to ensure that the reconstructed images match the acquired *k*-space measurements).^16^ λ is a weighting parameter controlling the balance between the data consistency term (the L_2_ norm term) and the sparsity term (the L_1_ norm term). In this work we used the first-order temporal finite differences as the sparsifying transform to minimize temporal total variation in the optimization. GRASP reconstruction was performed after grouping every 34 consecutive spokes as one contrast phase, resulting in a temporal resolution of ~3 seconds per temporal phase. All image reconstruction tasks were performed in software built in C++ and were implemented in a 64-core Linux server equipped with 256 GB memory. After reconstruction, images were interpolated to 40 partitions with an interpolated slice thickness of 3 mm. The reconstruction time for each 4D (3D+time) GRASP data was ~35–40 minutes.

### Perfusion Analysis

**FIGURE 1:**
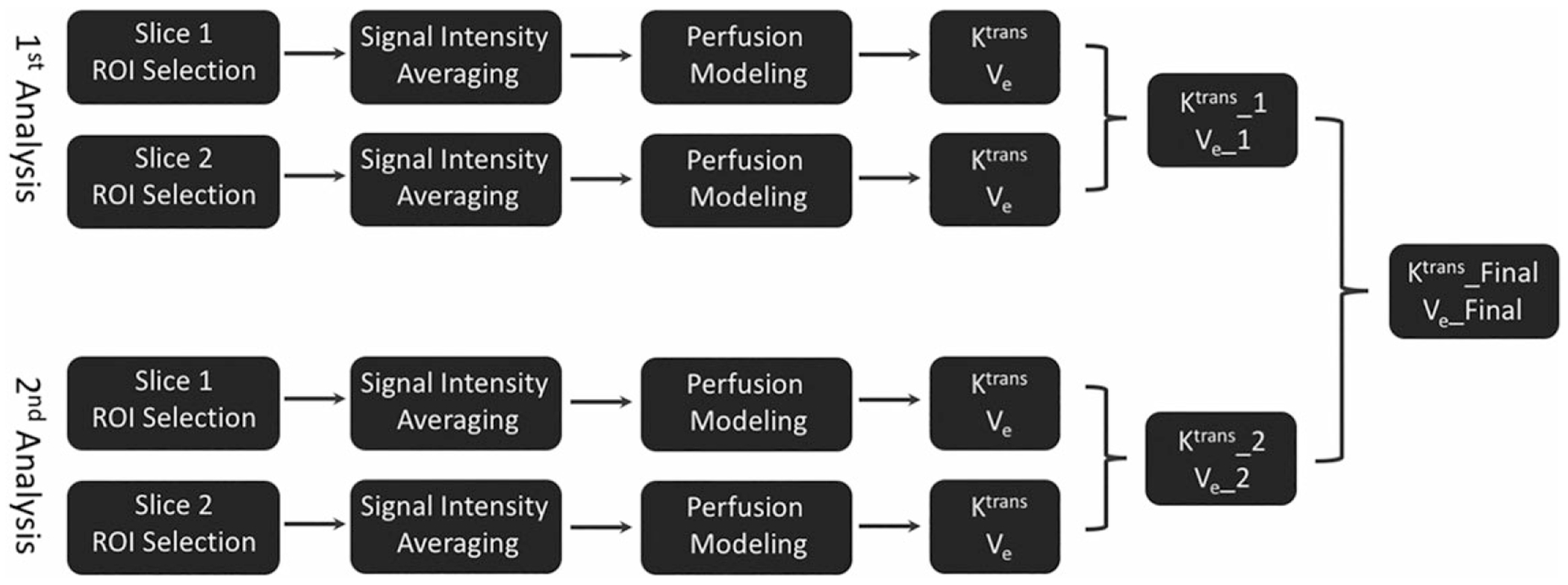
Overall workflow of the perfusion analyses. For each dataset, two ROIs were manually selected on two consecutive slices at a location matching the region where the tumor specimen was taken during the CT-guided biopsy. For each slice, the signal intensity of each pixel within the selected ROI was averaged together, and the averaged signal enhancement curve was fitted using a standard Tofts model to generate the K^trans^ and V_e_. The results from these two slices were averaged together as K^trans^_1 and V_e__1. This process was repeated after 3 days to generate K^trans^_2 and V_e__2. The results from two analyses were then averaged to yield the final K^trans^ and V_e_ (K^trans^_Final and V_e__Final), which were then compared with the corresponding MVD results.

**FIGURE 2:**
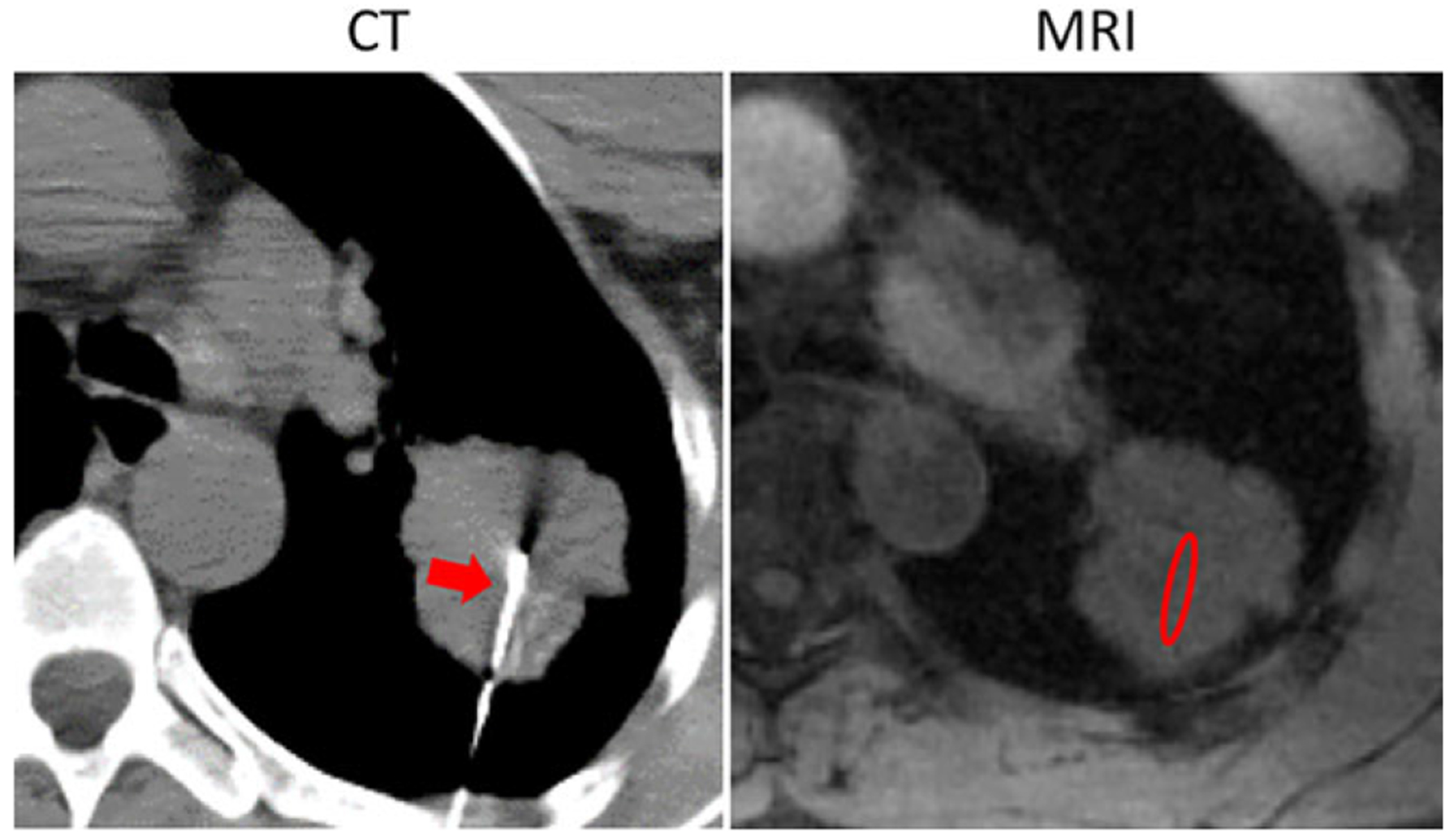
A representative example pair of CT and MR images showing that perfusion analysis was performed in an ROI matching the location where a tumor specimen was taken during the CT-guided biopsy. The red arrow in the left image shows the biopsy needle in the CT image and the red ellipse in the right image shows the region where an ROI was placed. (Note that the CT image was acquired with the patient in a prone position and the image was flipped to match the orientation of the MR image in this figure.)

Perfusion analysis was performed using an online image processing software (FireVoxel, available at https://wp.nyu.edu/firevoxel/downloads/). All analysis tasks were performed by a radiologist (L.C.) with 5 years of experience in clinical diagnostic MRI blinded to the pathologic findings. The perfusion analysis followed a procedure as previously described.^14^ The specific workflow is shown in Fig. 1. For each dataset, the radiologist (L.C.) manually selected two regions of interest (ROIs) on two consecutive slices at a location matching the location where the tumor specimen was taken during the Computed Tomography (CT)-guided biopsy. Figure 2 shows a representative example pair of CT and MR images, from which an ROI (red ellipse) was manually placed on the MR image to match the biopsy needle position in the CT-biopsy image. For each slice, the signal intensity of each pixel within the selected ROI was averaged together for improved signal-to-noise ratio (SNR), and thus fitting reliability, and the averaged signal enhancement curve was fitted using a standard Tofts model^17^ to generate the volume transfer coefficient (K^trans^) and the interstitial volume (V_e_). The K^trans^ and V_e_ values from these two consecutive slices were then averaged, shown as the K^trans^_1 and V_e__1 in Fig. 1. The arterial input function (AIF) for perfusion analysis was obtained from an ROI placed on the descending aorta, assuming a linear relationship between the contrast concentration and the signal intensity.

The analysis was repeated for all datasets by the same radiologist after 3 days to assess the measurement and remeasurement repeatability. Same as the procedure in the first analysis, the K^trans^ and V_e_ (indicated as K^trans^_2 and V_e__2 in Fig. 1) were calculated again. The results from two analyses were averaged to yield the final K^trans^ and V_e_ (referred to as K^trans^_Final and V_e__Final hereafter, as shown in Fig. 1), which were then compared with the corresponding MVD results.

To generate K^trans^ and V_e_ maps for demonstration purposes, perfusion analysis was also performed on a pixel-by-pixel basis for one slice in four cases with different pathological findings. For these analyses, perfusion fitting was performed without averaging the signal curves together, so that pixel-by-pixel K^trans^ and V_e_ maps can be generated. In addition, to show the stability of perfusion measurements, the K^trans^ and V_e_ were also obtained from the erector spinae muscle (normal healthy tissue). Corresponding results can be found in the Supplementary Materials.

### Histopathologic Analysis

All tumor specimens acquired during CT-guided biopsy were coated with standard marking ink (Hematoxylin-Eosin Staining Kit, Beyo-time, Jiangsu, China) and were fixed in 10% buffered formaldehyde for 24 hours before counting.

The tumor microvessels were highlighted by staining endothelial cells for CD34 with the use of a standard advin-biotin immunoperoxidase complex technique. The immunohistochemical slices were then analyzed by a pathologist (Y.W.) with 5 years of experience in clinical pathological diagnosis. A technique previously described by Weidner et al^18^ was used to identify microvessels that were delineated by CD34-positive cell. Each slice of the specimen was examined at a low-power magnification (×40) to identify the areas with the highest density of capillaries and small vessels. In each case, the most vascularized area was selected, and a ×200 field was stored as a JPEG file. Brown-staining endothelial cells that were clearly separated from other adjacent microvessels, tumor cells, and mesenchymal cells were counted as a single microvessel. Large vessels with lumens larger than eight red blood cells or thick muscular walls were excluded from the counts.

### Statistical Analyses

Pearson correlation coefficients were calculated to evaluate the repeat-ability in the perfusion measurements. The one-way analysis of variance (ANOVA) followed by the least significant difference (LSD)-t method of multiple comparisons were used to compare the quantitative perfusion results and the MVD results across different histologic types and tumor sizes, as well as nodal metastases status. Specifically, to compare results from different tumor sizes, patients were separated into two groups, including one group with tumor size >3 cm and the other group with tumor size ≤3 cm. To compare results from different nodal metastases status, patients were separated into another two groups, including one group with metastasis-positive and the other group with metastasis-negative. The metastases status was determined according to the 7^th^ edition of the TNM (tumor, node, metastasis) staging system for non-small-cell lung cancer. Pearson correlation coefficients were calculated to evaluate the correlations between the quantitative perfusion parameters and the MVD results. All statistical analyses were performed in SPSS (v. 18.0; Chicago, IL) and *P* < 0.05 was considered statistical significance.

## Results

The Pearson correlation coefficient was 0.983 (*P* < 0.001) and 0.972 (*P* < 0.001) for the measurement and remeasurement of K^trans^ (K^trans^_1 vs. K^trans^_2) and V_e_ (V_e__1 vs. V_e__2), respectively, showing good repeatability. The K^trans^_Final and V_e__Final from all the 25 lung patients were 0.33 ± 0.22 min^−1^ and 0.25 ± 0.12, respectively. Corresponding MVD in all the 25 patients was 49.68 ± 27.08 vessels/0.723 mm^2^. The K^trans^_Final, V_e__Final, and MVD were 0.36 ± 0.26 min^−1^, 0.27 ± 0.13, and 49.09 ± 29.84 vessels/0.723 mm^2^, respectively, in patients with adenocarcinoma. The K^trans^_Final, V_e__Final, and MVD were 0.34 ± 0.17 min^−1^, 0.26 ± 0.12, and 53.85 ± 23.53 vessels/0.723 mm^2^, respectively, in patients with squamous cell carcinoma. The K^trans^_Final, V_e__Final, and MVD were 0.13 ± 0.15 min^−1^, 0.14 ± 0.06, and 37.20 ± 28.28 vessels/0.723 mm^2^, respectively, in patients with small-cell carcinoma.

**FIGURE 3:**
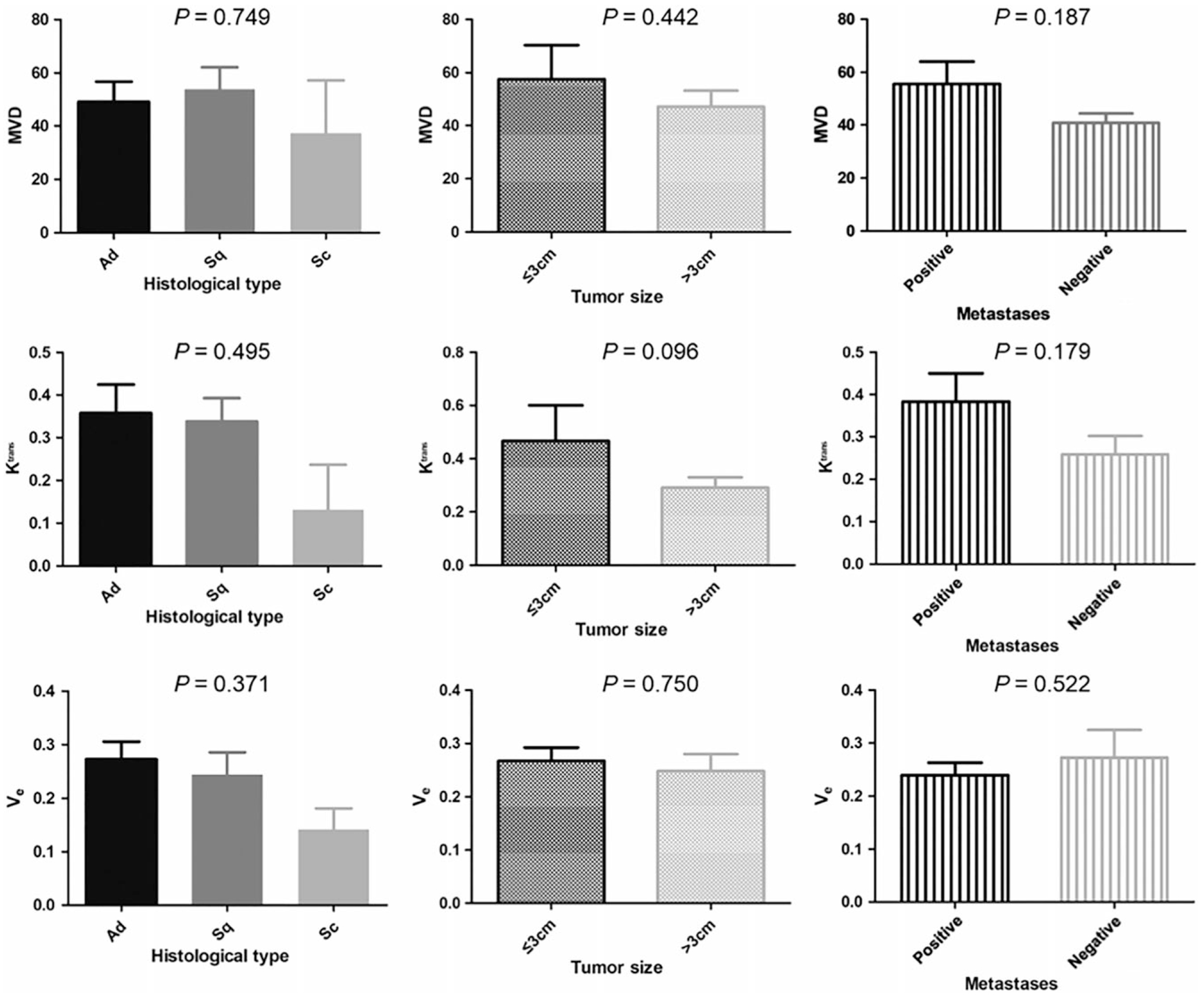
Summary of the K^trans^, V_e_, and MVD measured in different subgroups. The K^trans^, V_e_, and MVD were all higher in adenocarcinoma and squamous cell carcinoma than those in small-cell carcinoma without statistical significance. The K^trans^ and MVD from the >3 cm group were lower than those from the ≤3 cm group without statistical significance. Based on the nodal metastases status, the K^trans^ and MVD of metastasis-positive tumors were higher than those of metastasis-negative tumors without statistical significance. V_e_ is reported as a fractional measure of the interstitial volume. Ad: adenocarcinoma; Sq: squamous cell carcinoma; Sc: small-cell carcinoma.

Figure 3 shows the comparisons of perfusion results and the MVD across different histologic types, tumor sizes, and nodal metastases status. The K^trans^_Final, V_e__Final, and MVD were all higher in adenocarcinoma and squamous cell carcinoma than those in small-cell carcinoma, but statistical significance was not reached (*P* > 0.05, Fig. 3). The tumor size was >3 cm in 19 patients and was ≤3 cm in 6 patients. The K^trans^_Final and MVD from the >3 cm group were lower than those from the ≤3 cm group, but statistical significance was not reached (*P* > 0.05, Fig. 3). Based on the nodal metastases status, the K^trans^_Final and MVD of metastasis-positive tumors were higher than those of metastasis-negative tumors, but statistical significance was not reached (*P* > 0.05, Fig. 3). Please note that all the exact *P* values are labeled in Fig. 3.

**FIGURE 4:**
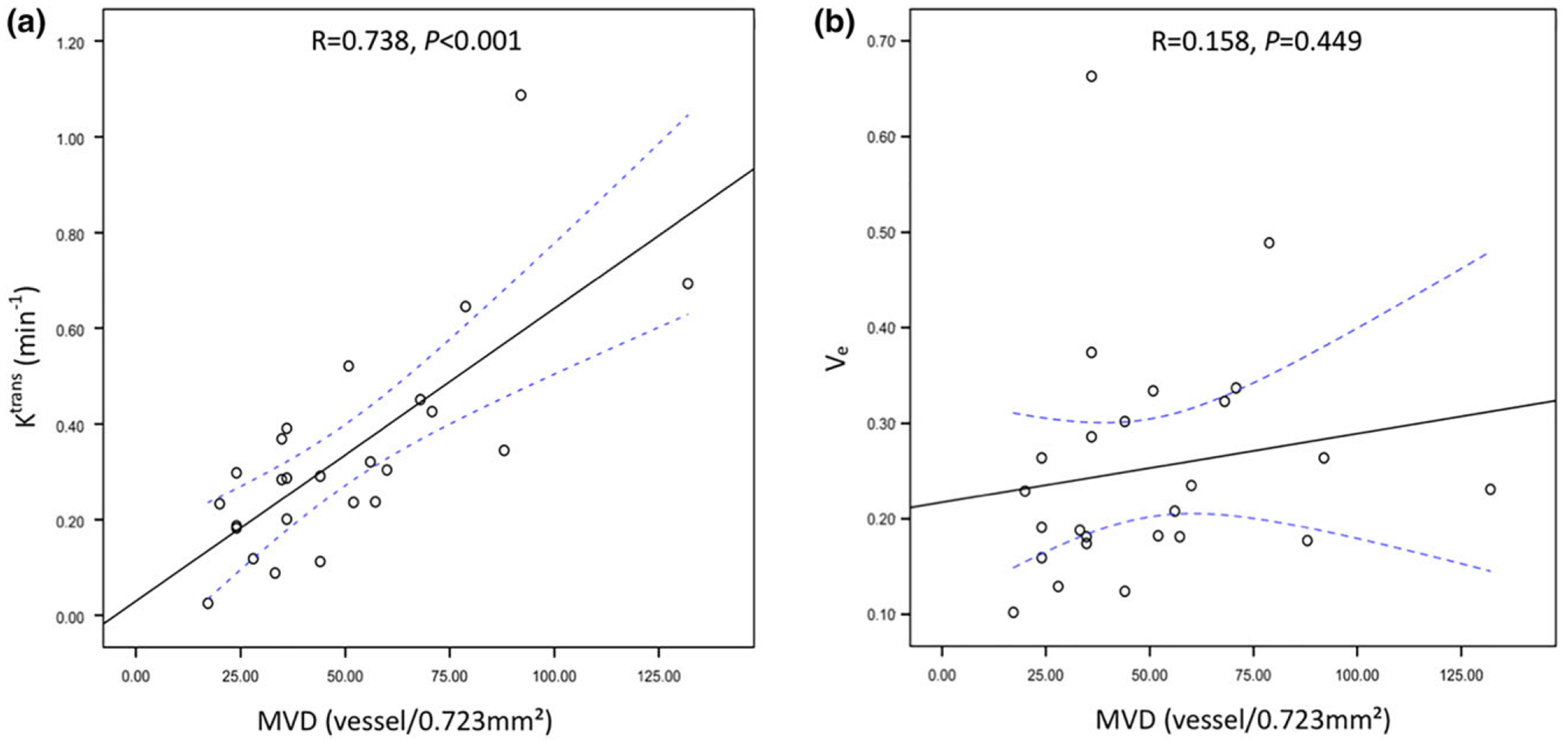
Scatterplots of the K^trans^ vs. MVD (left) and the V_e_ vs. MVD (right). There was a positive correlation between the K^trans^ and MVD (*R* = 0.738, *P* < 0.001). No significant correlation was found between the V_e_ and MVD (*R* = 0.158, *P* = 0.449). The solid black lines show the line of fit in linear regression and the dashed blue lines indicate the confidence interval.

Pearson correlation analysis showed that the K^trans^_Final and MVD were significantly correlated (correlation coefficient = 0.738, *P* < 0.001), as shown in Fig. 4a. However, no significant correlation was found between the V_e__Final and MVD (correlation coefficient = 0.158, *P* = 0.449), as shown in Fig. 4b.

**FIGURE 5:**
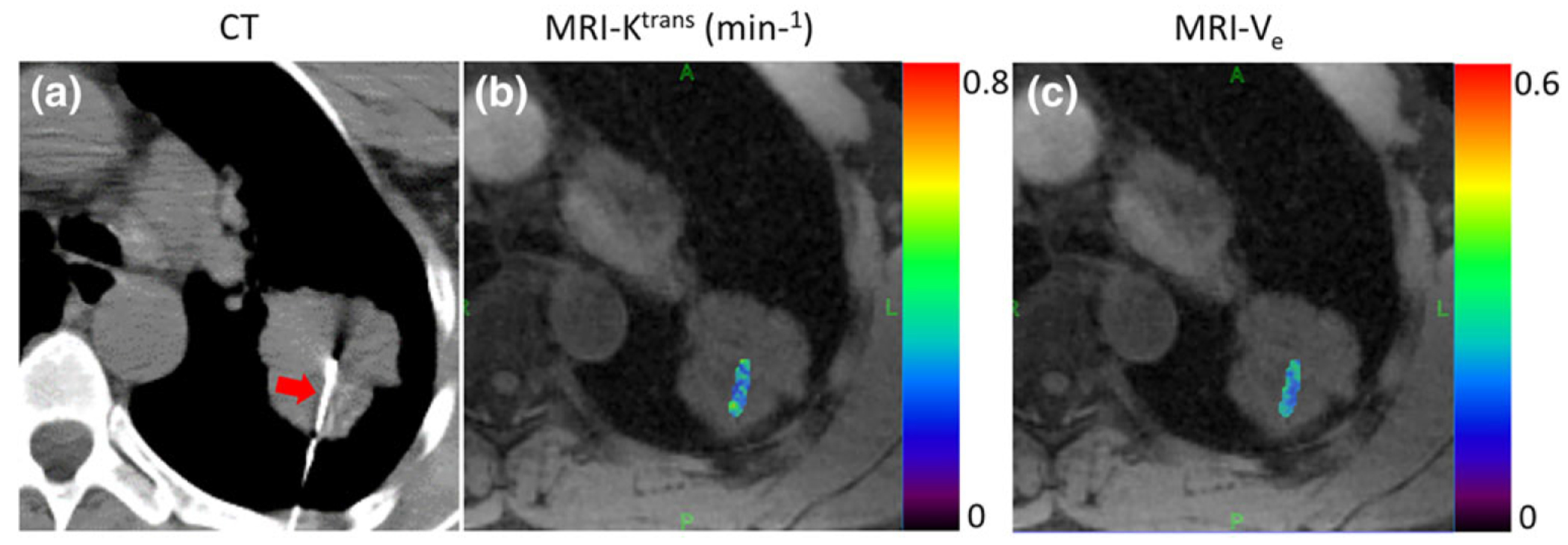
Regional K^trans^ and V_e_ maps **(b,c)** obtained from a patient with squamous cell carcinoma superimposed on a corresponding GRASP image. The ROI for perfusion analysis was selected using CT-biopsy image **(a)** as a reference, as previously shown in Fig. 2. The red arrow indicates the needle in CT biopsy.

**FIGURE 6:**
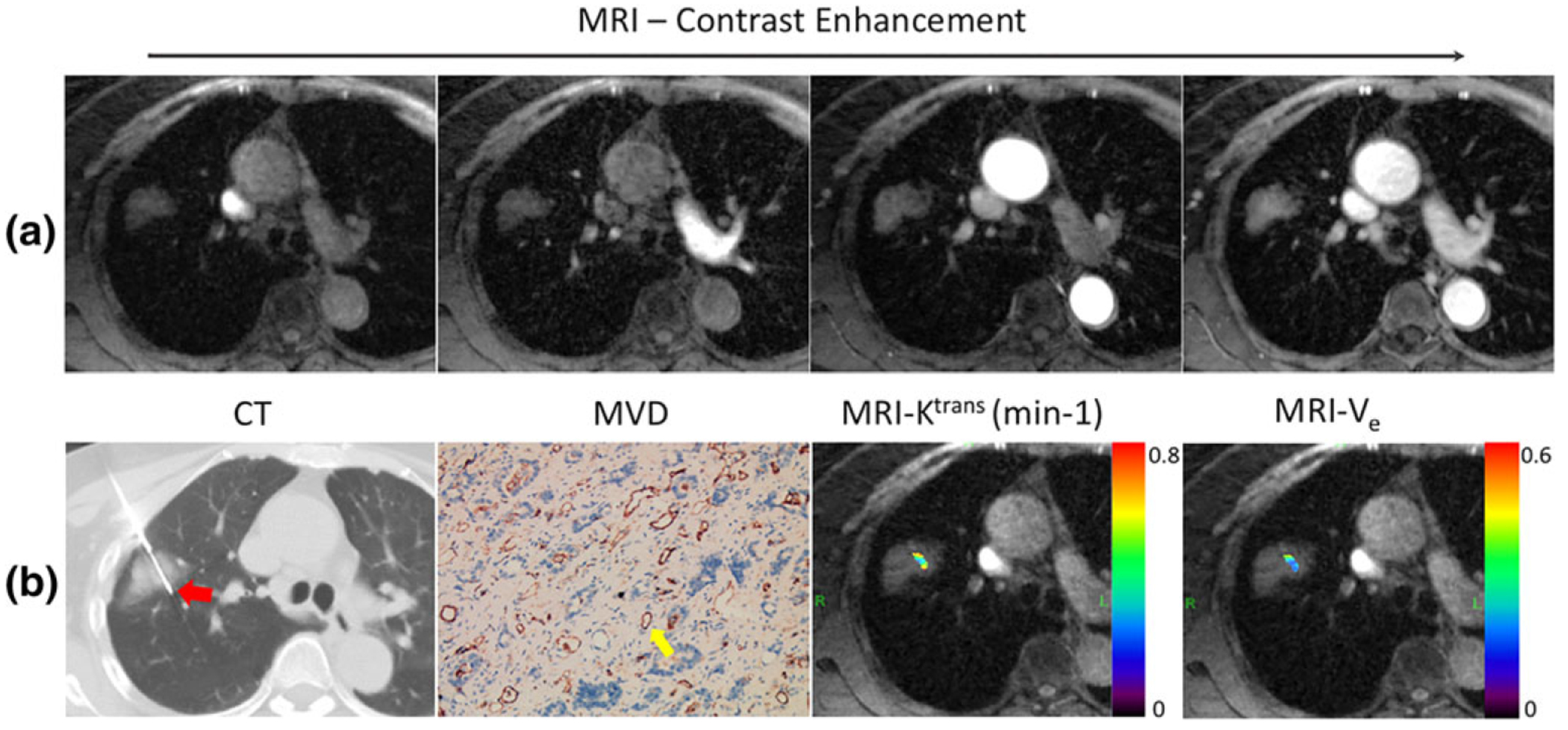
(a): Four different GRASP contrast phases from a patient with adenocarcinoma. (b): Corresponding CT-biopsy image, MVD image, and regional K^trans^ and V_e_ maps superimposed on a corresponding GRASP image. The yellow arrow indicates a microvessel and the red arrow indicates the needle in CT biopsy.

**FIGURE 7:**
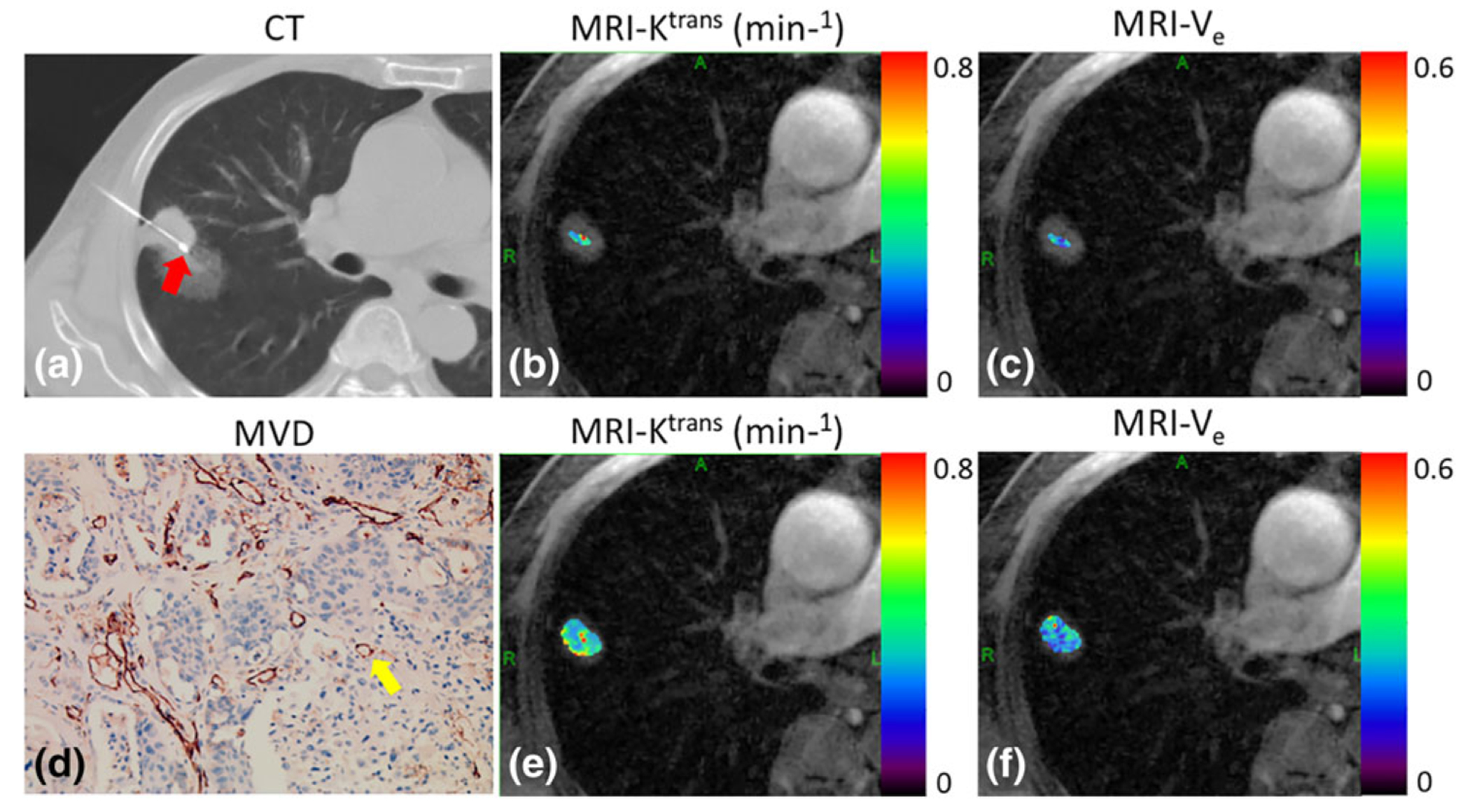
CT-biopsy image (a), MVD image **(d)** and both regional **(b,c)**, and whole-tumor **(e,f )** K^trans^ and V_e_ maps superimposed on a corresponding GRASP image from a patient with squamous cell carcinoma. The yellow arrow indicates a microvessel and the red arrow indicates the needle in CT biopsy.

**FIGURE 8:**
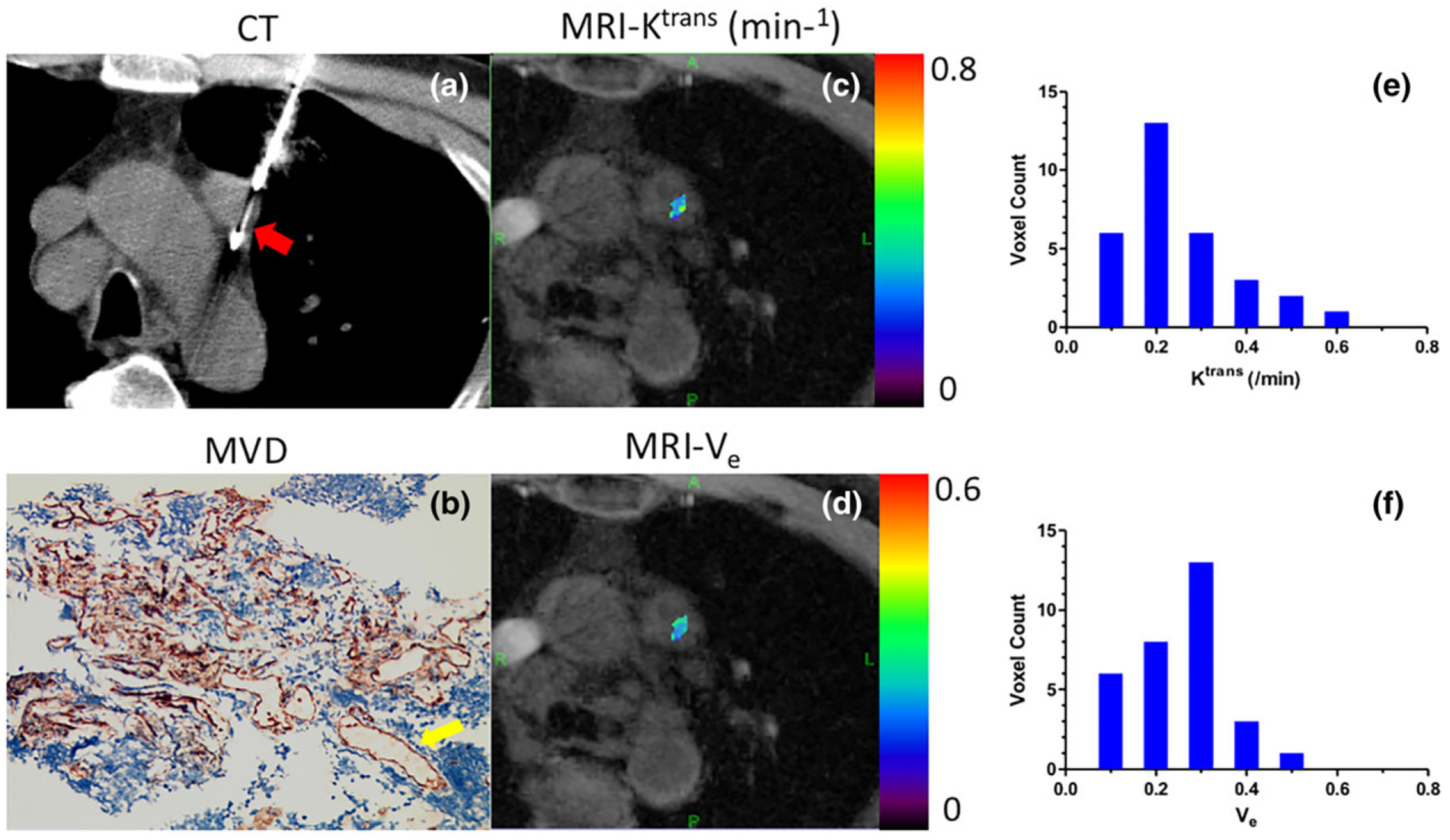
CT-biopsy image (a), MVD image **(b)**, and regional K^trans^ and V_e_ maps superimposed on a corresponding GRASP image **(c,d)** from a patient with small-cell carcinoma. Corresponding histograms of the K^trans^ and V_e_ maps **(e,f)** show the variation of the K^trans^ and V_e_ values from pixel to pixel. The yellow arrow indicates a microvessel and the red arrow indicates the needle in CT biopsy.

Figure 5 shows the regional K^trans^ and V_e_ maps (Fig. 5b,c) obtained from a patient with squamous cell carcinoma. The ROI was selected using CT-biopsy image (Fig. 5a) as a reference. Figure 6a shows four different contrast phases from a patient with adenocarcinoma. The corresponding CT-biopsy image, MVD image, and regional K^trans^ and V_e_ maps are shown in Fig. 6b. Figure 7 shows the CT-biopsy image, MVD image, and both regional and whole-tumor K^trans^ and V_e_ maps from a patient with squamous cell carcinoma. Figure 8 shows the CT-biopsy image, MVD image, and regional K^trans^ and V_e_ maps from a patient with small-cell carcinoma (Fig. 8a–d). Corresponding histograms of the K^trans^ and V_e_ maps are displayed in Fig. 8e,f, showing the variation of the K^trans^ and V_e_ values from pixel to pixel.

## Discussion

Tumor angiogenesis is known as a very important phenotype in malignant tumors. If this phenotype is activated, a variety of neovascularization patterns will be triggered, eventually leading to microvascular growth and increased blood supply in a tumor. This will in turn provide nutrition and oxygen for the growth, invasion, and metastasis of the tumor.^2,3^ Thus, assessment of tumor angiogenesis remains important in cancer study and therapy. It is known that the vascular structural abnormality in a tumor will result in enhanced vascular permeability, which increases the risk of tumor invasion and metastasis.^19^ Meanwhile, the heterogeneous distribution of tumor vessels leads to hypoxia, which in turn makes the tumor cells more invasive and metastatic.^20^ Therefore, the effect of antiangiogenic or vascular disruptive agents can be predicted from the characteristics of tumor vasculature reflected by perfusion measurements.

Several prior studies have already verified that perfusion parameters, such as the maximal enhancement rate and K^trans^ obtained from DCE-MRI, are related to tumor angiogenesis in animal models with lung cancer.^21,22^ However, although DCE-MRI has been widely used in routine clinical diagnosis, images are normally acquired with a relatively low temporal resolution for qualitative assessment only, which is primarily due to the limits of imaging speed in MRI and its high sensitivities to physiological motion in conventional imaging approaches. GRASP is a promising imaging technique to address these issues. It allows a continuous highly-accelerated data acquisition and enables both qualitative assessment and quantitative perfusion analysis simultaneously by reconstructing images with flexible temporal resolutions. GRASP has been successfully translated into routine clinical environment,^23^ and its performance has been tested in a range of clinical applications.^10^ In this study, we took one step further, to evaluate the correlations of the perfusion parameters obtained from GRASP MRI with MVD, and to investigate whether GRASP can be a potential alternative for assessing tumor angiogenesis.

Our experiments were performed in a cohort of patients with different subtypes of lung cancer. The preliminary finding of this study was the significantly positive relationship between MVD and K^trans^ obtained from the GRASP images. This verified our hypothesis that a stronger degree of perfusion indicates higher MVD in lung cancer. This preliminary result has good clinical significance, since it validated the perfusion measurement against current clinical standards for assessing tumor angiogenesis, suggesting that GRASP may be a useful free-breathing, noninvasive imaging technique for evaluation of tumor growth and cancer treatment response in tumor therapy. Meanwhile, it can overcome the limitation in the histological count of MVD, providing spatially resolved perfusion results to represent a whole tumor on a pixel-by-pixel basis. Our results also showed that V_e_ is not well correlated with MVD. A possible explanation for this finding is that the contrast agent distribution is usually assumed to be limited to the vascular plasma space and the extravascular extracellular space in a standard pharmacokinetic model, and the vascular volume is typically a small fraction of the total tissue volume less than 5%.^24^ As a result, the variance of V_e_ may be less than the variance of the MVD.

Prior research based on histopathology indicates that different types of lung cancer have different levels of tumor angiogenesis.^25,26^ To justify this, this study also compared the perfusion results and the MVD between different histological types of lung cancer. Our results showed that although the K^trans^, V_e_, and MVD are higher in adenocarcinoma and squamous cell carcinoma compared to small-cell carcinoma, the differences were not statistically significant. This finding was consistent with the conclusions of prior studies on DCE-CT perfusion.^27,28^ Meanwhile, our results also showed that K^trans^ and MVD in lung tumors with a size ≤3 cm were higher than those with a size >3 cm, but the differences were not significant either. We noticed that Li et al previously showed that perfusion parameters (perfusion index, peak intensification intensity, and blood flow) obtained from DCE-CT were significantly higher in lung tumors ≤3cm than the perfusion parameters in lung tumors >3 cm.^27^ This different finding between our study and Li et al’s study might be due to the relatively small sample size in our experiments and it requires further verification with an increased patient number. Regarding the nodal metastases status, our results were consistent with that of Li et al,^27^ indicating that K^trans^ and MVD in lung tumors with metastases was higher than those in lung tumors without metastases, but without statistical significance.

While some previous studies confirmed a notable positive correlation between the tumor MVD and quantitative perfusion parameters (e.g., peak intensification intensity and blood flow),^27,29,30^ which were consistent with our results, some studies reported a negative relationship.^31^ We speculate that this difference might come from the fact that tumor angiogenesis is heterogeneous, and the standard method to measure the MVD of a tumor only selects the most hot-spot region of tumor vessels. Thus, if the selected tumor vascular area does not match well with the region used for perfusion analysis, it may result in a certain bias, or even contrary results. In order to ensure that the image region for perfusion analysis is consistent with the area for MVD counting, the ROIs used for the perfusion quantification in this study were selected using CT-guided biopsy images as a reference, as previously described.^32^

This pilot study has several limitations requiring discussion. First, our sample size was relatively small, and in particular, we only had two patients with small-cell lung cancer. Thus, future studies to include a larger number of patients would be necessary to further validate the conclusion obtained from the present study. Second, the perfusion analysis used in this study assumes a linear relationship between the contrast agent concentration and the signal intensity. Although a separate T_1_ map can further improve the accuracy of perfusion measurements, this requires additional cumbersome steps and it was previously demonstrated that this assumption is feasible and adequate for comparison of quantitative perfusion parameters across different applications.^33^ Third, this study only evaluated the correlation of MVD with quantitative perfusion parameters obtained in the biopsy region. This was performed for validation against the current clinical standard. Once this is verified, the perfusion quantification can be performed in the whole lesion in future. Fourth, the ROIs used for perfusion analysis were manually selected based on corresponding CT-biopsy images. This required good experience in the users to accurately identity the desired regions. A potential approach might be to perform core-gistration between the CT and MR images for better accuracy in ROI selection. However, registration of CT and MR images from a moving organ, particularly when the imaging parameters are different, is very challenging and requires a sophisticated and reliable algorithm. This will be explored in future works.

In conclusion, the preliminary results from this study have suggested that high temporal resolution DCE-MRI using the GRASP technique is a promising noninvasive alternative for quantitative assessment of tumor angiogenesis in patients with lung cancer. The relationship between the K^trans^ obtained from GRASP MRI and the MVD may also be extendable to other types of cancer.

## Supplementary Material

Supplementary Material

Additional supporting information may be found in the online version of this article.
