# Supplementary material for: A Study of the Correlation of Perfusion Parameters in High-Resolution GRASP MRI With Microvascular Density in Lung Cancer
Source: J Magn Reson Imaging. Author manuscript; Available in PMC 2026 May 20. (PMC13189685; doi:10.1002/jmri.26340)

In order to demonstrate the stability of perfusion measurements, the K^trans^ and V_e_ were also obtained from the erector spinae muscle (i.e., normal healthy tissue) in a total of 18 patients, including 8 patients with adenocarcinomas, 8 patients with squamous cell carcinomas, and 2 patients with small cell lung cancer. One-way ANOVA analyses followed by LSD-t method of multiple comparisons were used to evaluate the differences of the corresponding perfusion parameters between different subgroups, and no statistical differences were found (*P*>0.05). The results are summarized as follows.

| Parameters | adenocarcinomas | squamous cell carcinomas | small cell lung cancer | *P* value |
| --- | --- | --- | --- | --- |
| K^trans^ (min^-1^) | 0.050±0.019 | 0.046±0.012 | 0.045±0.021 | 0.873 |
| V_e_ | 0.085±0.014 | 0.086±0.034 | 0.085±0.035 | 0.995 |

The supplementary figure below shows representative K^trans^ and V_e_ maps obtained from an ROI (red arrow) in the erector spinae muscle from a case with squamous cell carcinoma. The maps are superimposed on a corresponding GRASP MR image.


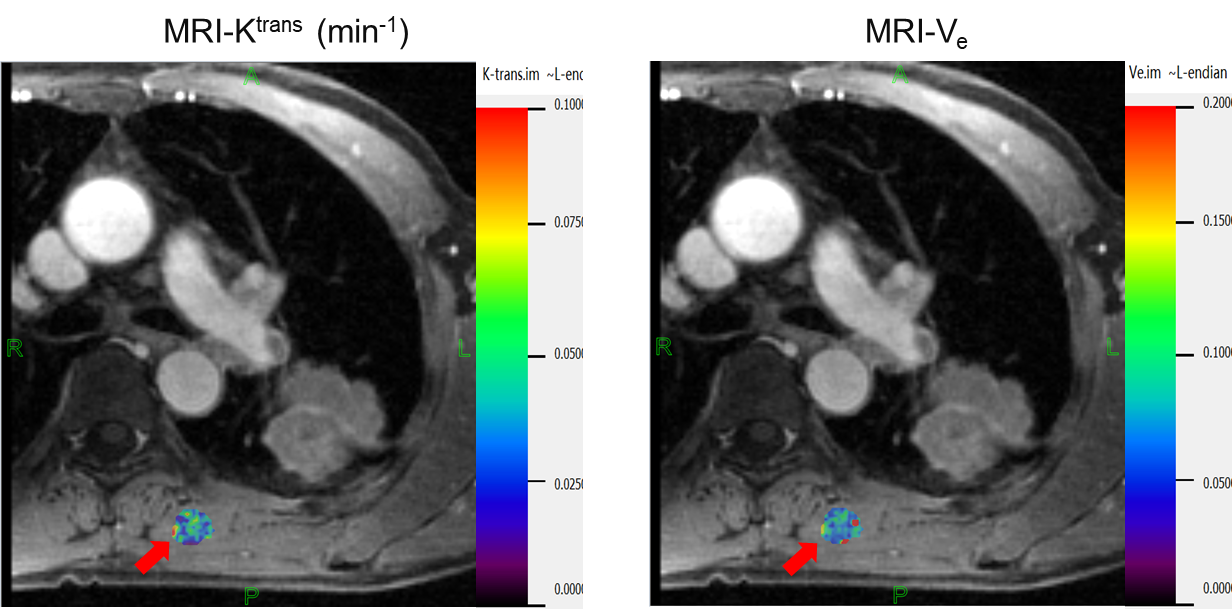

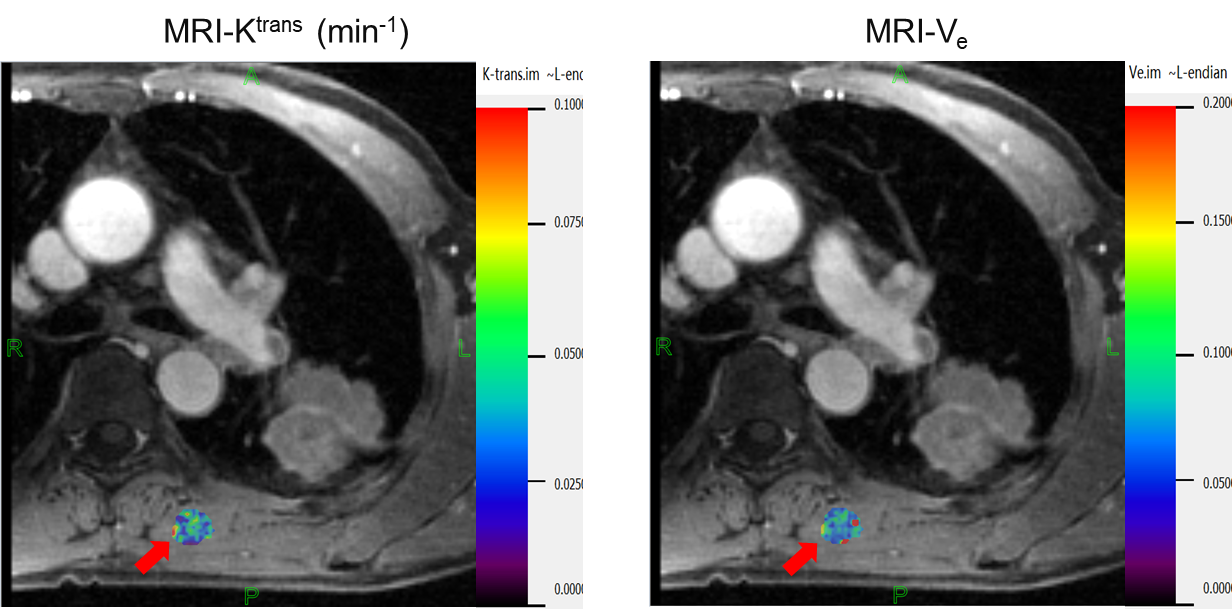

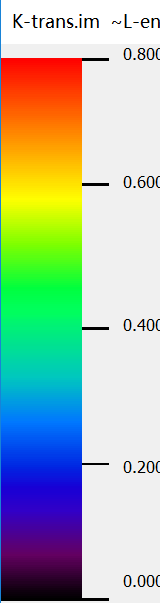


0.1

0

0.2

0


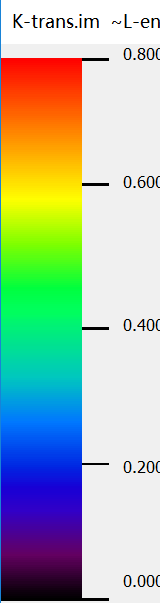

Supplement: Supplementary Material [file NIHMS2172370-supplement-Supplementary_Material.docx]
